# Burnout, resilience, and perception of mindfulness programmes among GP trainees: a mixed-methods study

**DOI:** 10.3399/bjgpopen20X101058

**Published:** 2020-07-29

**Authors:** Petra Hanson, Amy Clarke, Manuel Villarreal, Majid Khan, Jeremy Dale

**Affiliations:** 1 Warwick Medical School, University of Warwick, Coventry, UK; 2 School of Pharmacy, Centre for Behavioural Medicine, University College London, London, UK; 3 The Unit of Academic Primary Care, University of Warwick, Coventry, UK; 4 University of Albert Einstein, Mexico City, Mexico

**Keywords:** GP wellbeing, mindfulness, burnout, psychological, general practice

## Abstract

**Background:**

Trainee GPs are at risk of developing burnout as a result of high stress levels. Improving resilience may prevent the negative effects of stress on wellbeing, morale, and patient care, thereby supporting recruitment to general practice.

**Aim:**

To explore experiences of stress and burnout among GP trainees, and their level of interest in undertaking a mindfulness programme.

**Design & setting:**

A qualitative study was performed with a cohort of GP trainees in Coventry and Warwickshire.

**Method:**

This mixed-methods study utilised a survey with validated measures to investigate the prevalence of burnout, state of wellbeing, and resilience in GP trainees. Focus groups were also used to explore experiences of stress and burnout, and perceptions of mindfulness practice.

**Results:**

In total, 47 (response rate 39%) trainees completed the survey and 14 participated in focus groups. There was a high prevalence of disengagement (*n* = 36; 80%) and emotional exhaustion (*n* = 35; 77%), with 29 (64%) scoring above the cut-off value for both. While 16 (34%) reported already practising mindfulness, 39 (83%) described interest in engaging in mindfulness practice. The focus groups identified a range of issues relating to how trainees recognise stress and burnout, their help-seeking and coping strategies, the perceived barriers to practising self-care, and motivations for participating in mindfulness training.

**Conclusion:**

This study confirms the degree of stress and burnout that GP trainees experience, and their desire for greater wellbeing and resilience support. It identified a high level of interest in attending a mindfulness programme, but also barriers to engagement. Results of this research shaped the Mindful Practice Curriculum programme, which was later provided to this cohort of trainees.

## How this fits in

GP trainees experience high levels of stress, which is affecting their work–life balance and future career intentions to work in the NHS. Mindfulness programmes could improve trainees’ resilience and wellbeing, and thus promote more positive mental health, but evidence for this is lacking. Detailed understanding of trainees’ perceptions of barriers and motivators to attend such programmes will help shape an intervention that will be feasible, sustainable, and effective in the longer term.

## Introduction

GPs in the UK are known to experience high stress levels, and this is contributing to low morale, exhaustion, and burnout.^1,2^ Burnout affects the quality of patient care.^2,3^ It can also be harmful to doctors’ lives,^4^ as well as having a negative impact on healthcare delivery,^5^ and is contributing to the workforce crisis. It is an international issue that affects doctors at all stages of their career.^6–8^ There is evidence that burnout affects trainee and early career doctors twice as frequently as more senior doctors,^9^ which may reflect that those who suffer burnout leave the profession early.

A recent survey of doctors at the point of completing GP vocational training found over half (54.5%) reported having experienced work–life balance problems during their training; for most, this was influencing their future career intentions, with 27.1% of those experiencing problems expecting to have left NHS general practice within 5 years.^10^ Another survey found that of GPs who had completed specialist training within the previous 5  years, 41% would not have chosen to become a doctor on reflection, 34% regretted having chosen general practice as their specialty, and 36% were considering moving abroad to work.^11^

It is, therefore, important to consider ways of promoting wellbeing and resilience early on in training in order to provide trainees with the skills to cope with the pressures of being a GP. A systematic review of interventions to improve psychological wellbeing of primary care physicians concluded that *‘* *t* *here is an urgent need for high* *quality, controlled studies in GP well* *being* *’*.^12^ Resilience training for doctors has become increasingly popular and is recognised as a *‘* *potential tool for tackling* *current* *challenges in primary care* *’*.^13^ One such approach is mindfulness practice, with participating GPs describing that it enabled reconnecting with personal values and recentring.^13^ Mindfulness is defined as a capacity for enhanced and sustained moment-to-moment awareness of one’s own mental and emotional state and being, in the context of one’s own immediate environment.^14^ There is evidence that mindfulness practice among doctors improves patient-centred care, resilience, wellbeing, self-awareness, and intrapersonal skills.^15–17^ However, improving resilience does not only apply to the individual, but also to the team and wider organisation as a whole, and therefore should not be forgotten.^18^

The aim of the study was to explore experiences of stress and burnout among GP trainees, and their interest in undertaking a mindfulness programme. The objectives were to explore trainees’ current state of wellbeing, resilience, and burnout, and their previous experiences, knowledge, attitudes, and beliefs about mindfulness. This research was part of a larger study assessing the feasibility of delivering the Mindful Practice course to GP trainees.

## Method

This mixed-methods study utilised a survey to investigate the prevalence of burnout in a cohort of GP trainees and used focus groups to explore experiences of burnout, coping strategies, and perceptions of the relevance of mindfulness practice. The Consolidated Criteria for Reporting Qualitative Studies was used to report this part of the study.^19^ The study was undertaken between October 2018 and April 2019.

### Participants

All 120 second- and third-year GP trainees in Coventry and Warwickshire were invited to participate in the survey.

Permissions were gained from the general practice vocational training leads to enable investigators (PH and MV) to deliver a brief presentation on the study as part of the training programme and to hand out study promotion materials to trainees. Participants interested in taking part in the focus groups were asked to contact PH or MV by email for further details. Focus groups were held at a convenient time and location so as to not impact on training schedules.

### Data collection

#### Survey

The survey was made available for online completion using Qualtrics Survey Software. The survey included three validated questionnaires: The Oldenburg Burnout Inventory^19^ (to assess burnout); Smith and colleagues’ Brief Resilience Scale^20^ (to assess resilience); and Warwick–Edinburgh Mental Wellbeing Scale^21^ (to assess wellbeing). Other questions in the survey included exploring the form and mode of participants' mindful practice. Trainees were also asked open questions of other ways their wellbeing could be supported and how burnout could be reduced (Table 1). Survey data were collected anonymously.

**Table 1.** Open-ended questions

Do you think there is a need for provision of more wellbeing support to GP trainees in the region?
Would you like to receive training in resilience skills?
Would you be willing to engage with mindfulness practice?
In what other ways would you like to get support improving your wellbeing and reducing burnout?
Do you practise mindfulness in any form?

The survey was sent to eligible trainees via an email. Three reminders were sent. Trainee champions (those trainees who were interested in helping in this study) also reminded their colleagues about the survey and encouraged its completion.

#### Focus groups

Two focus groups were conducted with 14 participants in total. Focus groups lasted between 50 and 55 minutes. Topic guides were developed from the literature and through discussion, and covered areas such as: personal wellbeing and stress; coping strategies; knowledge and experience of mindfulness and resilience; and barriers and motivators to engaging with a mindfulness intervention as part of vocational training. Investigator AC (a research fellow with a background in health psychology) facilitated the focus group sessions, while investigators PH (a clinical academic fellow) and MV (a GP and mindfulness trainer) supported the sessions and took notes. Focus groups were audio-recorded using an encrypted device, uploaded to a secure server, and professionally transcribed verbatim. To protect anonymity, any identifiable information was omitted from the transcripts. The topic guide is shown in Table 2.

**Table 2.** Topic guide

What does the term personal wellbeing mean to you?What strategies or techniques have you used to improve your personal wellbeing or manage stress?Where did you find information about these strategies?
Do you think that strategies or techniques for coping with work-related stress should be considered as a part of specialist training?If so, how and when should this be delivered?If not, what alternatives should be provided to help GP trainees deal with stress?
What do you understand by the terms 'resilience' and 'mindfulness'?What experience, if any, have you had of training or practice related to these?
What would attract you to participate in a programme aimed at increasing mindfulness and resilience?What concerns would you have, if any?What could be done to overcome these?

#### Quantitative analysis

Two components (emotional exhaustion and disengagement) were used to assess the prevalence of burnout.^20^ Mean scores of each component were calculated (and reverse scoring applied when necessary). Cut-off scores of ≥2.25 for exhaustion and a score ≥2.10 for disengagement were used to predict problematic burnout,^21^ and burnout was indicated if both scores were above the given values.

Smith and colleagues’ Brief Resilience Scale consists of six items, three of which are reverse scored. The overall score is average of the six items. The score for Warwick–Edinburgh Mental Wellbeing Scale was calculated by adding all scores together.

#### Qualitative analysis

Transcripts were entered into NVivo (version 11) to facilitate data analysis. An inductive thematic approach to analysis was undertaken to identify themes at a semantic level following the steps of Braun and Clarke.^22^ A single investigator (AC) generated the initial codes and collated codes into potential themes. Preliminary themes were shared and discussed with investigators PH, MV, and JD, all of whom had read and familiarised themselves with the transcripts. Agreed themes were then refined and applied to the remaining dataset, and then refined during the writing phase of analysis.

## Results

Forty-seven trainees completed the survey (response rate 39%) and 14 participated in a focus group. All of the participants were currently employed in general practice or a hospital-based training post. The survey was completed anonymously. To protect identities, sociodemographic data and information about their training year was not collected. The focus group participants included six men, and eight women.

The Warwick–Edinburgh Mental Wellbeing Scale was completed by 47 trainees. The average wellbeing score was 46.7 (standard deviation [SD] = 7.32). This is lower than the national average wellbeing score of 51.6, based on *Population Norms in Health Survey for England Data*.^23^

Smith and colleagues’ Brief Resilience Scale was completed by 46 trainees. The average score was 3.02 (SD = 0.65), which is at the lower level of the normal population range (normal is 3–4.30).^24^

Below, the quantitative and qualitative data are drawn on to report on the key themes, which related to: stress and burnout; support and other coping mechanisms; perceived barriers to practising self-care and attending a mindfulness intervention; and perceived motivations to practising self-care and attending a mindfulness training intervention.

### Stress and burnout

Exemplar quotations for the theme ‘stress and burnout’ are shown in Table 3.

**Table 3.** Exemplar quotations from participants: stress and burnout

**Major themes**	**Minor themes**	**Exemplar quotations**
Stress and burnout	Knowledge and training	*'But I don’t know how much it’s been expressed that you, as the doctor, you need to look after yourself.'* (Focus Group 2, R7)
		*'… we’ve not been trained to do little more than gather a history that can possibly identify someone’s suicide risk or whether they’re psychotic. Like we just need to figure it out ourselves in terms of our training.'* (Focus Group 1, R2)*'I wouldn’t have said that I’d had any training on how to cope or how to get through terrible things…'* (Focus Group 2, R2)
	Keeping up appearances	*'…we have to appear to cope. We have to pretend all the time that we think we know exactly what’s happening or what’s going on and what to do next. “Trust me, I’m your doctor,” …but half the time inside you’re going, “Oh, I can’t remember that,” or, “I don’t know what to do.” But yet we, we cope. What do we do with all that stress? Where does all that mental energy go?*' (Focus Group 1, R5)
	Detaching from work	*'My mum, she was GP and she used to tell me, “When you leave the job, leave the work, you just shut the door, so you leave everything in your practice, don’t take it home.” But it’s not easy.'* (Focus group 2, R4)

Forty-five trainees completed the Oldenburg Burnout Inventory. There was a high prevalence of disengagement, with 36 (80%) trainees scoring above the cut-off level of 2.10 (mean = 2.36; SD = 0.4). Thirty-five trainees (77%) scored above the cut-off level (2.25) for emotional exhaustion (mean = 2.68; SD = 0.44). Twenty-nine (64%) participants scored above the cut-off value for both, indicating a high risk of burnout.

#### Knowledge and training

Participants acknowledged the importance of their own mental wellbeing and were able to provide a clear definition of burnout. However, the importance of self-care was not something that they had experienced as a priority within the vocational training curriculum. Clinical training was deemed inadequate to support trainees in coping with increasingly complex, mental health-focused patient consultations and challenging aspects of the role such as managing patient complaints. Participants acknowledged that internalising complaints was unhelpful for their mental health, yet many of the group struggled with this and reported no training in this area.

#### Keeping up appearances

The perceived lack of skills or coping mechanisms were seen to negatively impact on clinical practice and personal risk of burnout. Participants perceived that there was a professional duty to hide feelings of inadequacy and instead maintain appearances of seeming in control.

#### Detaching from work

Participants also felt that they lacked training in how to detach from work at the end of the day. Instead, many experienced a tendency to take on patients’ problems and resulting compassion fatigue from the perception that *'* *it’s give, give, give* *…* *'* (Focus group 2, R7).

### Support and coping mechanisms

Exemplar quotations for the theme ’support and coping’ mechanisms are shown in Table 4.

**Table 4.** Exemplar quotations from participants: support and coping mechanisms

**Major themes**	**Minor themes**	**Exemplar quotations**
Support and coping	Threshold for seeking help	*'You are, you know, you are the doctor… so you keep everything for yourself and that’s, that’s the, the biggest* [issue]*, you know, the threshold to seek help….'* (Focus group 1, R6)
		*'I think there’s a stigmatisation amongst doctors that if you need to take more time, if you need to have some extra support, that, overall, that’s perceived negatively*.' (Focus group 2, R2)
		*'Quite often you get asked, “What do you think is going on then?"... And you make your own diagnosis.'* (Focus Group 1, R2)
	Recognising burnout in the professional culture of medicine	*'I found that times where I haven’t been happy, but it’s taken me quite a while to realise that. And actually it wasn’t till kind of about six months nearly down the line, I thought, “Actually, no, things aren’t right and, and this is what’s wrong in life and this is what’s making me unhappy.”'* (Focus group 1, R2)
		*'No one should be going to work and being made to feel, you know, belittled or disrespected. And I think, at times, in medicine, I have certainly experienced it.'* (Focus group 2, R2)
	Maladaptive coping approaches	*'They avoid it, they might do some exercise but they might watch loads of Netflix, might not go out, might smoke, might drink, might take drugs.'* (Focus group 1, R5)
	Adaptive coping approaches	*'…pretty much every day actually, I’ll go home and there’ll be something that’s happened that day that I’ll talk to my wife about who’s not medical … it might help me focus on a bit more of how I felt at the time or how the person felt at the time and that can be really helpful … she’ll often put her non-medical spin on it which I think can be quite good.' (*Focus Group, R5)
	Personal acceptance	*'I know, for me, it’s kind of my acceptance of my own limitations, like accepting that I’m not perfect, which took a really long time to learn…*' (Focus group 2, R6)
	Use of mindfulness apps	*'I was trying to do things like downloading mindfulness apps and trying to actively do mindfulness. And then I find that, “Oh, I’m not stressed any more, don’t bother with that anymore,” and it just goes out the window*.' (Focus group 1, R5)

Responders to the survey showed a desire for greater support with most (*n* = 39; 83%) expressing a need for a greater wellbeing support as part of the vocational training curriculum, and most (*n* = 40; 85%) also indicating a desire to receive training in resilience skills. A minority (*n* = 16; 34%) reported practising mindfulness (in the form of yoga, meditation, use of mindfulness apps, or mindfulness videos).

#### Seeking help

Participants described the high threshold at which they would seek help, and recognised this as being problematic. They described feeling reluctant to seek help from family, peers, senior colleagues, and their own healthcare providers. Within the family environment, participants still felt they maintained the role of doctor and were more likely to keep things to themselves. Participants felt increased pressure to hide struggles around times of transition, but overall described the ongoing fear that asking for help would be stigmatised within a profession that was deemed by others as challenging by nature. A further barrier that some described was a perception that they were treated differently by doctors when seeking help. They described being asked to suggest their own diagnosis, which was felt as a barrier for asking for psychological support.

#### Recognising burnout in the professional culture of medicine

The high threshold for which participants would seek support often meant delaying action until a point of crisis. This was partly owing to failure to recognise early signs of burnout or changes in emotional states. A professional culture fuelled by high expectations, whereby stress is normalised, is likely to contribute to a lower emotional awareness and reluctance to seek help as it can be *'* *perceived as a negative attribute* *'* (Focus group 2, R2). Some participants described how supervisors were a good source of support, but others described instances of being reprimanded for speaking about difficulties. Participants had experienced senior colleagues who belittled concerns and made unhelpful distinctions between their own experiences and what it takes to be a trainee today, reinforcing feelings of inadequacy. However, participants felt that more needed to be done to encourage and support people within the medical profession to look out for each other to avoid increasing levels of burnout.

#### Maladaptive coping approaches

Owing to the difficulties described in seeking help from others, participants often sought more individual-based approaches to coping. Participants described various strategies, some of which were maladaptive; this included avoidance of the situation, isolating themselves from social situations, as well as escapism through recreational drugs.

#### Adaptive coping approaches

More positive strategies included seeking support outside of the profession from family or friends. Gaining a different perspective was seen as useful, but most felt that self-care strategies that could be identified and actioned by the individual without support from others were optimal. Some participants targeted their actions to particular stressors using strategies such as getting outdoors more, or meditation and audio sounds to assist with sleeping or problems switching off.

#### Personal acceptance

For most participants, personal acceptance of limitations and striving to view themselves with a non-judgmental lens was important. Strategies to assist with this included trying to be better organised, self-help books, and the use of mindfulness apps.

#### Use of mindfulness apps

Mindfulness apps were viewed positively by those who had used them. However, barriers to continued use reflected perceptions of feeling 'cured' from stress and no longer needing it, and the expiry of free app subscriptions.

### Perceived barriers and motivations to practising self-care and attending a mindfulness intervention

Exemplar quotations for the theme ‘perceived barriers and motivations to practising self-care and attending a mindfulness intervention’ are shown in Table 5.

**Table 5.** Exemplar quotations from participants: perceived barriers and motivations to practising self-care and attending a mindfulness intervention

**Major themes**	**Minor themes**	**Exemplar quotations**
Barriers	Concept of mindfulness	*'I find mindfulness an odd concept because it’s probably something that people didn’t think about 20 years ago* *.'* (Focus group 1, R5)
		*'I might talk about mindfulness or something as a strategy to manage a patient and it wouldn’t be the first time where I’ve seen somebody of an older age roll their eyes, you know, as a response to something that I’d suggested to a patient.'* (Focus group 1, R4)
	Fear of introspection	*'… there might be people who are on the cusp of being in a really, really bad mental state, who might be that, you know, the swamp underneath, paddling like crazy just to get through. And those people might avoid doing something like this because they think if they just open the box a little bit, it might spill out.'* (Focus group 1, R2)
	Time	*'I agree with that, I think it, I think a lot of us are aware of strategies that might help, but it’s about having time to put those into place with busy working lives, home lives.'* (Focus group 2, R2)
Motivators	Desired benefits	*'I’m hoping it will improve my mental state, that it will give me more tools to better cope with life, my own life and my patients’ lives and make my kids ... my wife sort of like, she can take care of herself, that’s what I’m hoping.'* (Focus group 1, R4)
	Necessity for mindfulness training when working	*'I think it* [resilience training] *should be more, once you’re actually working in the environment you work in, it would be more useful, but if it’s kind of put* [to the test] *dealing with, how to deal with complaints, how to ... it’s kind of facing, “That stuff’s going to happen, this is how you deal with it.”'* (Focus group 2, R6)

Most (*n* = 39 trainees; 83%) survey responders expressed interest in engaging in mindfulness practice. The main barriers to such engagement were perceived as being time constraints and lack of evidence for mindfulness interventions.

#### Barriers

##### The concept of mindfulness

In the focus groups, while mindfulness was generally viewed positively by those who had previously experienced it, some participants felt it was *'* *wishy* *washy* *'* (Focus group 2, R6) and an odd concept. Some reported that mindfulness for clinical practice was not taken seriously by more senior colleagues. Such perceptions were deemed as barriers that would need to be addressed to facilitate interest for attending a mindfulness programme. Participants emphasised that for such a programme to be attractive to trainees, mindfulness would need to be presented as an evidence-based process, 'taught in a very scientific and clinical manner' (Focus Group 1, R4).

##### Fear of introspection

Other barriers that were recognised included the fear of getting in touch with personal feelings about distress and stress, *'* *if they just open the box a little, it might spill out* *'* (Focus group 1, R2).

##### Time

Finding time for themselves in the midst of busy working and home lives was perceived as a barrier to engaging in mindfulness. Different work situations, such as being on call or early morning surgeries, were seen as potential barriers to initiating regular mindfulness practice. However, some participants described how at points of 'crisis' they had found the time to engage in things such as mindfulness apps.

#### Motivations

##### Desired benefit

The participants shared a desire to enhance their skills to better manage stress in order to feel more in control, achieve a better work–life balance, and improve their overall mental state. Others took the view that participating in mindfulness training was useful to completing their training portfolio.

##### Necessity for mindfulness training when working

Many felt that, regardless of the extent to which resilience skills training occurs at undergraduate level, it is important to receive such training when working as a doctor in the clinical environment, and that ongoing follow-up and support should be available.

## Discussion

### Summary

This study identified a high prevalence of disengagement, emotional exhaustion, and overall burnout among GP trainees. Most participants were interested in developing skills to improve stress management and resilience, and would like to see this incorporated into their vocational training. Perceived barriers to taking part in a mindfulness programme were: lack of evidence to support mindfulness practice in a postgraduate setting, constraints of time, and the perceived stigma within the profession associated with help-seeking. Having a structured approach to delivery of such a programme, together with protected time, were viewed as facilitators to engaging with mindfulness training. Additionally, it was recognised that the skills learnt from such training carried both professional and personal benefits. Overall, participating in a mindfulness training programme was viewed positively and seen as something that would add value to current vocational training.

### Strengths and limitations

A key strength of this study is the use of validated survey measures of burnout and wellbeing with the use of qualitative methods to understand what trainees experience as the aspects of their working life that are contributing to stress and burnout. While the response rate (39%) to the survey was relatively low, this is not unusual for this type of study. As such, the sample may not be fully representative, and it only represented views of trainees in one sub-region of England, which may be different to those training in other areas. The high prevalence of burnout among the cohort might indicate that doctors who completed the survey may be those most at risk of burnout and low wellbeing. However, the opposite could also be true, in that doctors who suffer from greater burnout are less likely to complete surveys, resulting in underestimation of the overall prevalence. Another limitation is that sociodemographic information was not collected and so the representativeness of the survey or focus group participants cannot be commented on. Using focus groups for exploration of trainees’ views was beneficial as participants could challenge each other and themes could be explored in greater depth. However, it is possible that participants may have been unwilling to share some personal views within the context of a focus group of peers.

### Comparison with existing literature

Reported levels of burnout among medical professionals vary depending on specialty, country of work, and length of practice. In the US, it was found that burnout affected 50% of surgical trainees and 76% of internal medicine trainees.^25^ High prevalence of burnout (75%) was found among newly qualified medics in Australia.^8^ A recent General Medical Council survey identified 37% of trainees as having low levels of burnout and 23.8% with high levels of burnout,^26^ a prevalence that is broadly in line with the participants in the present study. However, the participants in the present study had a low level of resilience, which is at odds with previous findings of a higher than average resilience among doctors.^23^ Whether this finding applied to GP trainees more generally needs further investigation.

### Implications for research and practice

This study provides insight into the stress and burnout experienced by GP trainees, and highlights the need for greater support in training. It has identified trainees‘ perceptions of the barriers and motivators to engaging with mindfulness practice, which is the first step to successful implementation of mindfulness programmes as part of vocational training. Structured mindfulness training has produced positive results among physicians in the US, Australia, and Brazil,^25,27,28^ but more studies are needed to increase the body of evidence and determine the best mode of delivery in the context of GP vocational training. Additionally, personal resilience training should be linked with improving resilience at the organisational and team level.^18^ Findings from this study have already provided useful information that influenced delivery of the Mindful Practice Curriculum to GP trainees, and the effect this had on burnout and resilience will be reported in a future article.
